# Pulling the BAX trigger for tumor cell death

**DOI:** 10.18632/oncotarget.24201

**Published:** 2018-01-12

**Authors:** Denis E. Reyna, Evripidis Gavathiotis

**Affiliations:** Evripidis Gavathiotis: Department of Biochemistry, Department of Medicine, Albert Einstein Cancer Center, Albert Einstein College of Medicine, Bronx, NY, USA

**Keywords:** BAX, BCL-2, mitochondria, MOMP, apoptosis

Cancer cells evade cellular death pathways, such as apoptosis, in order to ensure an uncontrollable growth as well as resistance to various treatments. Dysregulation of the BCL-2 family of proteins that critically regulate the intrinsic apoptotic pathway contributes to the pathogenesis of cancer [1]. A major mechanism of cancer progression relies on the overexpression of anti-apoptotic BCL-2 proteins, such as BCL-2, BCL-X_L_ and MCL-1, which steers cellular fate towards survival [1]. Anti-apoptotic members bind and neutralize the BH3 domains of multi-domain pro-apoptotic BCL-2 members, BAX and BAK, and pro-apoptotic BH3-only proteins such as BIM and BID, thereby suppressing cell death [1]. Often, more resistant tumors to cancer therapeutics concomitantly suppress or degrade pro-apoptotic BH3-only proteins limiting the proteins available to directly activate BAX and BAK to promote apoptosis [2]. Inhibitors of anti-apoptotic BCL-2 proteins targeting specific members have been developed with promising clinical results; however, their use has been limited in cancers that rely on additional anti-apoptotic mechanisms for survival [1, 2]. These findings suggest the need for alternative therapeutic strategies that have the potential to overcome such blockades of tumor cell death.

Pro-apoptotic BAX is a critical member of the BCL-2 protein family. Following apoptotic stimuli, activating BH3-only proteins interact with the trigger site on BAX resulting in a series of structural changes that culminate in the translocation of BAX from the cytosol to the mitochondria outer membrane (MOM) [3, 4]. Once anchored to the MOM, BAX oligomerizes and creates a pore where apoptogenic factors, such as cytochrome *c*, escape and activate the caspase cascade that orchestrates apoptosis. In the vast majority of cancer cells, BAX is expressed in an inactive conformation or suppressed by anti-apoptotic proteins [1, 2]. Mutations in BAX that may cause its inactivation are present at low frequency in different tumors [5]. Therefore, we hypothesized that targeting direct activation of BAX to promote apoptosis may offer an alternative therapeutic strategy for cancer.

**Figure 1:**
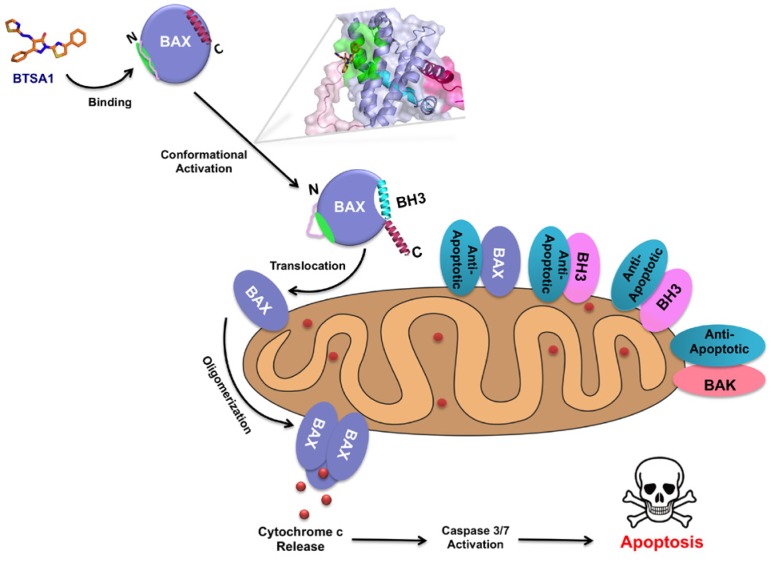
Direct BTSA1-mediated BAX activation induces apoptosis BTSA1 binds the trigger site (green) of cytosolic BAX monomer and induces BAX conformational activation that includes α1-α2 loop (pink) opening, α9 helix (red) mobilization and α2 helix (BH3) (cyan) exposure. Conformationally active BAX anchors to the outer mitochondrial membrane using α9 helix and upon further conformational changes and oligomerization, BAX induces mitochondrial outer membrane permeabilization and apoptosis.

To assess the therapeutic potential of direct BAX activation, we set out to discover small molecules that mimic the BAX-activating interactions of BIM with the N-terminal trigger site of BAX [6, 7]. We used a pharmacophore model and medicinal chemistry to rationally design BAX trigger site binders based on previous structural models of the BIM BH3 helix and BAM7 compound. Synthesized compounds were optimized based on a binding assay that evaluates competition of fluorescein-labeled BIM BH3 helix from the BAX trigger site. We identified BAX trigger site activator 1 (BTSA1), a lead compound with high affinity and selectivity for the BAX trigger site against other anti-apoptotic BCL-2 proteins [7]. Moreover, using biochemical BAX activation assays we demonstrated that binding of BTSA1 to BAX induced all the necessary conformational changes that lead to BAX translocation and oligomerization to promote MOM permeabilization (Figure 1).

We investigated the cell-based capacity of BTSA1 to induce apoptosis in a panel of acute myeloid leukemia (AML) cell lines that overexpress various anti-apoptotic proteins. BTSA1 promptly and robustly decreased cellular viability in a dose and time-dependent manner. BAX translocation to the OMM, cytochrome *c* release and activation of effector caspases were significantly accomplished at 4 hrs (Figure 1). The pro-apoptotic effect of BTSA1 was correlated with the protein levels of BAX and not of the anti-apoptotic BCL-2 proteins. Cellular specificity was confirmed with a weaker analogue of BTSA1, a pharmacological inhibitor of BAX mitochondrial channel and a siRNA knockdown of BAX. A biotinylated form of BTSA1 was synthesized for pull down experiments demonstrating engagement with cellular cytosolic BAX consistent with binding of BTSA1 to the BAX trigger site. *In vivo*, BTSA1 suppressed the growth of two human AML mouse xenografts, as evidenced in bone morrow, peripheral blood and liver samples of treated mice, which lead to an increased mouse survival. Toxicological analysis with similar dosing schedule showed no obvious toxicity in several tissues, blood counts and hematopoietic stem cells and progenitors. Consistently, BTSA1-induced BAX activation selectively promoted apoptosis in primary AML blast cells and pre-leukemic stem cells from patients compared to healthy hematopoietic stem cells and progenitors. The selective activity of BTSA1 correlated with higher BAX expression in AML patients compared to healthy controls. Furthermore, in AML patients, BCL-2 is expressed in higher levels compared to healthy controls. Thus, BTSA1 and Venetoclax co-treatment resulted in significantly synergistic pro-apoptotic activity in several AML cell lines.

In summary, we have identified BTSA1 as a potent and selective BAX trigger site activator [7] (Figure 1). Binding of BTSA1 promotes BAX activation leading to mitochondrial dysfunction and activation of the apoptotic pathway. The impact of BTSA1-induced BAX activation in AML cells is regulated by the protein levels of BAX and availability of anti-apoptotic BCL-2 to sequester activated BAX. Our findings provide proof-of-concept for BAX as a therapeutic target in AML and suggest that direct BAX activation is well tolerated *in vivo*. Lastly, BTSA1 has favorable drug-like properties and will be a useful compound in future studies to assess the therapeutic potential of direct BAX activation in other cancer models as a single agent or in combination with other cancer therapeutics.
